# Molecular Mechanisms and Pathophysiology of Acute Stroke: Emphasis on Biomarkers in the Different Stroke Subtypes

**DOI:** 10.3390/ijms23169476

**Published:** 2022-08-22

**Authors:** Teresa Gasull, Adrià Arboix

**Affiliations:** 1 Cellular and Molecular Neurobiology (CMN) Research Group at Germans Trias i Pujol Research Institute, 08916 Badalona, Barcelona, Spain; 2 Cerebrovascular Division, Department of Neurology, Hospital Universitari del Sagrat Cor, Universitat de Barcelona, 08029 Barcelona, Catalonia, Spain

According to WHO data, strokes are the second leading cause of death in adult males, the first cause of death of adult women worldwide and one of the most important causes of disability and dementia in adults [1], with up to 50% of patients being left chronically disabled, which has a huge impact on health care and economics; impact that is expected to increase due to population aging [2].

Stroke has a complex pathophysiology. The knowledge of the pathophysiological mechanisms of stroke and its analysis based on the study of its different etiological subtypes is essential to contribute to a better understanding of both its natural history and the therapeutic possibilities to improve cerebral reperfusion, neuroprotection and secondary prevention [3,4].

Increasing evidence suggests that the brain is exquisitely sensitive to even short-duration ischemia and that multiple mechanisms are involved in the tissue damage that results from cerebral ischemia. Ischemic stroke initiates a cascade of events, including ATP depletion, ionic dysregulation, the increased release of glutamate and the excess production of free radicals, as well as edema and inflammation; all these events eventually contribute to cell death. In contrast, in intracerebral hemorrhage, oppression and destruction of brain tissue by hematoma is the primary cause of brain injury, but inflammation, coagulation response and the toxicity of the released hemoglobin play a pivotal role as well. Cell death after ischemic stroke has been attributed in the past mainly to necrosis or apoptosis, but recent reports show the involvement of other newly described forms of cell death [5].

This Special Issue is aimed at understanding some of the most novel pathophysiological and molecular aspects of acute stroke and at providing a critical overview of the underlying factors involved in stroke-related brain injury, emphasizing mainly the most promising protein, genetics and epigenetic biomarkers. This Special Issue includes ten papers [6,7,8,9,10,11,12,13,14,15], two original articles and eight review papers (Table 1).

A total of six articles analyze cerebral infarctions [6,9,11,12,14,15], one intracerebral hemorrhage [13], one subarachnoid hemorrhage [7] and two analyzed globally stroke [8,10]. Emphasis is also placed on the different etiological subtypes, assessing lacunar-type cerebral ischemia [9], atherothrombotic infarcts [6,12], spontaneous intracerebral hemorrhage [10,13], subarachnoid hemorrhage [7] and on post-stroke consequences, including vascular-type cognitive impairment [8] and post-stroke lung damage [15].

Andone et al. [6], in a systematic review, analyzed the role of biomarkers strongly related to atherothrombotic stroke and identified 23 biomarkers strongly related to atherothrombotic stroke and assessed their roles as risk factors, detection markers, predictors and therapeutic targets. The authors remark that biological biomarkers offer new data that could improve clinical practice but require additional neuroimaging markers to accurately detect or diagnose atherothrombotic stroke.

In a review paper, Wang et al. [7] summarized the recent literature reporting the role of microRNAs as biomarkers in aneurysmal subarachnoid hemorrhage (aSAH) management, a hemorrhagic stroke subtype with high mortality. Biofluid miRNAs hold great potential as biomarkers for aSAH and its complications (such as delayed brain injury by cerebral vasospasm, hydrocephalus and re-bleeding). Targeting miRNAs to achieve therapeutic benefits in aSAH complication management is an attractive perspective, and the identification of miRNA biomarkers should provide insights into the mechanistic roles of miRNA in aSAH.

In a systematic review, Kim et al. [8] evaluated proteins in blood, especially related to brain damage and post-stroke cognitive impairment. Vascular-type cognitive impairment is the second leading cause of dementia and is more common in the concomitant presence of brain atrophy in neuroimaging [16]. The authors reported that total cholesterol (TC), low density lipoprotein cholesterol (LDL-C), Homocysteine (Hcy) and C-reactive protein (CRP) could be useful potential biomarkers for the brain damage in stroke-associated with cognitive impairment.

Lacunar ischemic strokes are caused by small infarctions that occur in regions supplied by one perforating artery and represent from 11 to 27% of acute strokes, according to different series. Lacunar ischemic strokes are not only a prevalent type of stroke but could also be the first clinical manifestation of cerebral small vessel disease, which causes severe physical and vascular cognitive impairment in the long-term. Rudilosso S et al. [9] performed an updated review and offer a clinical perspective for the managing of lacunar strokes in light of the latest insights from imaging and translational studies. They concluded that more efforts are needed in the future to encourage the external validation of new cerebral small vessel disease (SVD) markers and include them in large multicenter prospective studies, including randomized clinical trials (RCTs). Improvement in the diagnostic work-up, including advanced imaging, is desirable in cerebral small vessel disease. Translational research is necessary to develop new treatments.

Cullell et al. [10], in their original article, performed a proteome-wide association study (PWAS) to integrate genomic and proteomic data in order to discover common mechanisms regulating the two main acute manifestations of small vessel disease: small vessel strokes (SVS) and non-lobar intracerebral hemorrhage (NL-ICH) [17]. Their results demonstrated the presence of an association at the proteomic level of ICA1L in the brain with SVS and non-lobar ICH. The association is conditioned by the cisregulation of its protein levels. ICA1L was previously identified in association with SVS and ICH in a genome-wide association study (GWAS) [18].

Aramburu-Nuñez et al. [11] summarized recent research investigations on the role of stress granules dynamics in ischemic stroke and remark that the study of the different molecules implicated in stress granules dynamics may have implications on a basic molecular level and also in the clinical application in stroke patients.

A review by Carballo-Perich et al. [12] provides an up-to-date summary of the state of knowledge on the epigenetic biomarkers of carotid atherosclerosis and plaque vulnerability. The identification of vulnerable plaque biomarkers that allows patients at greater and lesser risk of stroke to be distinguished will be extremely useful for decision making and the selection of patients for surgical or intensive medical treatment. The authors concluded that, at present, it is not possible to determine which carotid atherosclerotic plaques will become symptomatic. However, epigenetic biomarkers, and especially non-coding RNAs, are pointed at as promising biomarker candidates of plaque vulnerability.

Intracerebral hemorrhage (ICH) is a complex and heterogeneous disease and represents the final manifestation of different types of cerebral small vessel disease, usually categorized as: lobar (mostly related to cerebral amyloid angiopathy) and deep (due to hypertension-related vasculopathy). 

Giralt-Steinhauer et al. summarize, in their review [13], the current knowledge on the genetics and epigenetics of this devastating stroke subtype, while discussing how these new data contribute to the understanding of the ICH pathophysiology and to develop therapeutic strategies. The authors’ conclusion was that combining genomics information with epigenomics, transcriptomics, proteomics and metabolomics data offers a unique opportunity to enhance the understanding of the pathological processes related to ICH.

In their review, Gallego-Fabrega et al. [14] comment on several of the most relevant studies on the genetics and epigenetics of ischemic stroke and the potential use of the data generated by GWAS. The authors confirmed that the combination of GWAS data with bioinformatic analysis is a powerful tool to understand the biological mechanisms of complex diseases as ischemic stroke.

The original article by Faura et al. shows that experimental stroke increases the protein content in the bronchoalveolar lavage fluid (BALF), an effect that is associated to the differential expression of specific proteins in the BALF and in the lungs of ischemic mice, as assessed by two different techniques. Some of the differentially expressed proteins have a role in cell damage, and changes in the expression of these proteins are neither associated with the impairment of the alveolo-capillary barrier nor conditioned by the severity of ischemia-induced brain damage. These results are pivotal to target the increased risk or mortality and disability produced by stroke-associated pneumonia.

As reported in many previous studies and in this Special Issue, the mechanisms of acute stroke are complex. Understanding detailed mechanisms underlying acute stroke mainly in its different stroke subtypes may lead to the development of effective therapeutic strategies for the prevention of neuronal death, and for the improvement of functional recovery from neuronal injury.

## Figures and Tables

**Table 1:** Articles included in the present Special Issue “Molecular Mechanisms and Pathophysiology of Acute Stroke”.

Authors	I/H	Stroke Subtype	Topic	Characteristic of the Article	Conclusions
Andone et al. [6]	I	Atherothrombotic ischemic stroke	Protein/amino acid-related blood biomarkers in atherothrombotic stroke.	Systematic review	Authors identified 23 biomarkers, including C-reactive protein and homocysteine, strongly related to atherothrombotic stroke and assessed their roles as risk factors, detection markers, predictors and therapeutic targets. They could be used as additional tools for etiological diagnosis.
Wang et al. [7]	H	Aneurismal subarachnoid hemorrhage (aSAH)	MicroRNAs as biofluid biomarkers in aSAH management.	Update review	Biofluid MicroRNAs hold great potential as biomarkers for aSAH and its complications.
Kim et al. [8]	I + H	Post-stroke cognitive impairment	Blood proteins as biomarkers for stroke, especially related to brain damage and cognitive decline.	Systematic review	Homocysteine, C-reactive protein, total cholesterol and LDL-cholesterol could be possible biomarkers in patients with post-stroke cognitive impairment.
Rudilosso et al. [9]	I	Lacunar stroke	Neuroimaging and translational research	Update review	Improvement in the diagnostic work-up, including advanced imaging, is desirable in cerebral small vessel disease. Translational research is necessary to develop new treatments.
Cullell et al. [10]	I + H	Small vessel strokes (SVS) and intracerebral hemorrhage (ICH).	Proteome-wide association study (PWAS) to integrate the genomic and proteomic data to discover the two main acute mechanisms of SVS and ICH.	Article	Identification an association at the proteomic level of ICA1L with SVS and non-lobar ICH.
Aramburu-Núñez et al. [11]	I	Ischemic stroke	Stress granules dynamics	Update review	The study of the different molecules implicated in stress granules dynamics may have implications on a basic molecular level and also in the clinical application in stroke patients.
Carballo-Perich et al. [12]	I	Atheromatous ischemic stroke	Epigenetic biomarkers of plaque vulnerability in carotid atherosclerosis.	Update review	At present it is not possible to determine which carotid atherosclerotic plaques will become symptomatic. Non-coding RNAs are, specifically, promising biomarker candidates of plaque vulnerability.
Giralt-Steinhauer et al. [13]	H	Intracerebral Hemorrhage (ICH)	Genetics and epigenetics	Update review	Combining genomics information with epigenomics, transcriptomics, proteomics and metabolomics data offers a unique opportunity to enhance the understanding of the pathological processes related to ICH.
Gallego-Fabrega et al. [14]	I	Ischemic stroke	Genome-wide association studies (GWAS)	Update review	The combination of GWAS data with bioinformatic analysis is a powerful tool to understand the biological mechanisms of complex diseases as ischemic stroke.
Faura J et al. [15]	I	Ischemic stroke	Stroke-induced differential expression of specific proteins in lungs, brain and serum.	Article	Elucidates new molecules differentially expressed in the lung interphase after stroke and provides clues of new mechanisms underlying stroke-induced lung damage.

I: ischemic stroke/H: Hemorrhagic stroke.
